# A Case Report on a Rare Disease: Dyskeratosis Congenita

**DOI:** 10.14740/jocmr2056w

**Published:** 2015-03-01

**Authors:** Bethel Shiferaw, Satish Mukka, Lawrence Ha, Ebisa Bekele, Radhames Ramos De Oleo

**Affiliations:** a Department of Medicine, Nassau University Medical Center, East Meadow, NY, USA

**Keywords:** Dyskeratosis congenita, Hereditary disorder, Bone marrow failure, Dystrophic nails, Leukoplakia, Skin pigmentation, Telomere, Rare disease

## Abstract

Dyskeratosis congenita (DC) is a rare hereditary disorder characterized by bone marrow failure, cancer predisposition (11-fold increase compared to general population), ectodermal dysplasia (nail dystrophy, oral leukoplakia, and abnormal skin pigmentation) and other additional somatic abnormalities. A 22-year-old man presented with fever, chills, and a painful throat. Leukoplakia was noted on his tongue and some of his fingers and toe nails were markedly dystrophic. His skin seemed spotted with pigmentation on the anterior chest and neck. Patient reported family history of “blood disease” and leukemia. He was admitted for the management of neutropenic fever (102.9 °F, WBC: 940, ANC: 404, platelets: 21,000 and Hb: 9.2), and was put on broad spectrum antibiotics. A bone marrow biopsy revealed normocellular marrow with erythroid predominance and mild dyserythropoiesis. Chromosomal analysis indicated no numerical or structural chromosomal abnormalities. The fluorescence *in situ* hybridization report did not reveal any assay specific abnormalities. Viral marker for hepatitis and studies of autoimmune antibodies showed negative results. CT scan had shown splenomegaly. Patient was discharged after he has been fever and symptoms free, with a follow-up at hematology clinic. Individuals with DC most commonly present with skin pigmentation, dystrophic nails and leukoplakia, as presented in this case. Genetic abnormality was not confirmed in our patient, but it is suggested that X-linked recessive pattern would be significant because of greater prevalence in men than in women (10:1). The distribution of blood counts and bone marrow biopsy categorizes our patient in the moderate aplastic anemia class which is the most prevalent pattern. When fever in young patients with a failing bone marrow is diagnosed, it is important that physicians rule out the possible underlying causes. DC is a rare disease, but can be diagnosed by simple inspection of the mucocutaneous abnormalities. DC is a severe multisystem disorder associated with premature morbidity and mortality. We believe that reporting this case would add more information to the existing fund of knowledge.

## Introduction

Dyskeratosis congenita (DC) is a rare hereditary disorder characterized by triad of nail dystrophy, oral leukoplakia and abnormal skin pigmentation [1]. Other presentations include bone marrow failure, a predisposition to malignancy and fatal pulmonary complications [1]. The leading cause of death in patients with DC is bone marrow failure that will develop in around 85% of cases and is responsible for 80% of the observed mortality [2].

First described in the medical literature in 1906, DC was originally thought to be a skin disease that also affects the nails and the mouth. Only later in the sixties was it realized that patients with these skin changes develop bone marrow failure. The prevalence of classic DC is approximately 1/1,000,000 individuals, with 200 cases reported in the literature [3].

The way that healthcare providers diagnose and treat DC is changing quickly as researchers learn more. However, the immunologic features of DC remain under diagnosed and under treated despite the fact that immunodeficiency is a major cause of premature mortality in DC [4].

Bone marrow failure is considered to be an important and major complication of DC. We report on a case of young man with DC complicated by neutropenic fever, in an attempt to increase awareness about this rare disease.

## Case Report

A 22-year-old Hispanic man presented to our hospital because he was feeling very sick. Few days earlier, he suffered from fever, chills, and painful throat which were worsening. He felt easily exhausted but his weight or appetite was not changed. He has headache but no neck stiffness. He has no cough, bowel or urinary compliant. He has no travel history or similar symptoms in the family. He has never been exposed to poisonous gas or radiation. He has taken no medications for his illness or other reasons. He does not smoke, drink alcohol or use illicit drugs. He works as a clerk. He has siblings but they do not have any skin pigmentation or nail dystrophy. He had an uncle (maternal side) with similar illness who eventually died of leukemia.

**Figure 1:**
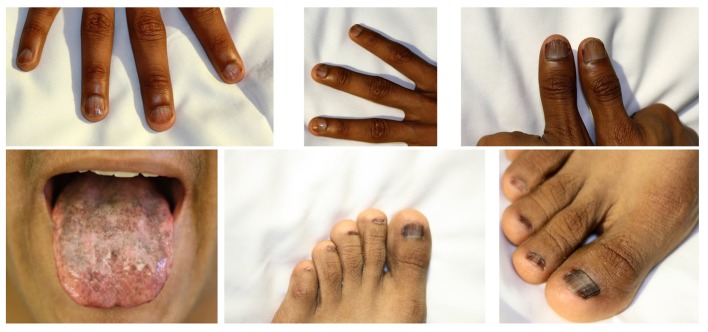
Pictures of the patient showing nail dystrophy and leukoplakia.

Leukoplakia was noted on his tongue and some of his finger and toe nails were markedly dystrophic (Fig. 1). His skin seemed spotted with pigmentation on the anterior chest and neck. Auscultation of the lungs revealed clear breath sounds bilaterally without wheezes or rhonchi. The heart beat sounded regular without murmur.

On the day of his admission, his blood pressure was 106/74 mm Hg, pulse rate was 106/min, respiratory rate was 20/min, and body temperature was 102.9 °F. His WBC count was 940 with 37% neutrophils (ANC: 404), platelets 21,000 and Hgb 9.2. Organ function tests were within normal ranges. He was admitted with possible diagnosis of neutropenic fever, and was put on broad spectrum antibiotics.

The source of infection was not identified but blood/urine culture and chest X-ray were ordered. The results showed negative blood culture, urine culture grew Staphylococcus and Lactobacillus bacterial species, and chest X-ray indicated hilar enlargement after which CT of the chest showed no abnormality. Antibiotic was continued but sore throat still persisted and antifungal was added on the second day which resolved the sore throat eventually. Other results include electrocardiogram showing no abnormality, CT scan of head and neck soft tissue indicating no sign of tumor or malignancy.

When he was 14 years old, he had vague symptoms and went for checkup, at that time, pancytopenia was noted. During his hospital follow-up, he received bone marrow study, chromosomal analysis, fluorescence *in situ* hybridization (FISH) and other tests. His latest bone marrow biopsy revealed normocellular with erythroid predominance and mild dyserythropoiesis. Moderate aplastic anemia without dysplasia was diagnosed throughout his hematologic evaluation and follow-up. He was not a candidate for bone marrow transplantation because marrow failure was not that severe. Chromosomal analysis indicated no numerical or structural chromosomal abnormalities. The FISH report did not reveal any assay specific abnormalities. Viral marker for hepatitis B or C that could cause pancytopenia showed no specific viral infection. Studies of autoimmune antibodies showed negative results. The CT scan had shown splenomegaly. We noticed that he had progressive bone marrow failure which has put him at risk for life-threatening complications (Table 1).

**Table 1:** Hematologic Testing Results for the Patient at Different Ages

Study	Age 20	Age 22	Age-appropriate values at age 22
WBC	2.79	0.94	4,500 - 11,000/µL
Hemoglobin	13.4	9.2	12 - 16 g/dL
Platelets	47.3	21	140,000 - 400,000/µL
ANC	1,510	404	1,500 - 8,000
ALC	872	319	2,500 - 3,500

He was discharged after he has been fever and symptoms free, with an appointment to at hematology clinic. Unfortunately patient failed to keep his follow-up with the clinic.

## Discussion

DC is characterized by a triad of dystrophied nails, skin pigmentation, and leukoplakia. It can be associated with bone marrow failure (pancytopenia) as presented in this case. The skin pigmentation and nail changes usually appear first, before the age of 10 years, and then bone marrow failure develops often before the age of 20 years, which was characteristically seen in our patient [5]. Genetic abnormality was not confirmed in our patient, but it is suggested that X-linked recessive would be significant because of higher prevalence in men than in women with a ratio of 10:1 [3].

Studies over the last 10 years have demonstrated that DC is principally a disease of defective telomere maintenance. DC patients have very short telomeres and the genetically characterized cases of DC have mutations in different genes which encode components of the telomerase complex [5]. To date, CTC1, DKC1, TERC, TERT, TINF2, NHP2, NOP10, and WRAP53 are the genes in which mutations are known to cause DC and result in very short telomeres. Mutations in one of these eight genes have been identified in approximately half of individuals who meet clinical diagnostic criteria for DC [6].

Mutations in telomerase cause excessive telomere attrition, which leads to premature cell death and chromosome instability that eventually either reduces/exhausts the stem cell reserve, thereby leading to clinical features such as BM failure [7]. In our case, the distributions of blood counts categorize the patient in the moderate aplastic anemia class and these findings are the features of the most usual pattern.

Another major cause of death is malignancy occurring mainly on mucosal surfaces showing leukopenia. Therefore, it is vital to recognize the increased risk of upper aero-digestive tract cancers in these patients (11-fold increase compared to general population) [8]. Cognizant of this fact, we have looked for possible head and neck malignancy in our patient for whom the results ruled out any tumor or malignancy at this point.

When fever in young patients with failing bone marrow is diagnosed, it is important that physicians rule out the possible underlying causes. DC is a rare disease, but it can be diagnosed by simple inspection of the mucocutaneous abnormalities. The classic triad of abnormal fingernails and toenails, reticular pigmentation of the neck and upper chest, and oral leukoplakia is diagnostic [6, 9]. Patients who meet the clinical diagnostic criteria should be investigated further. Therefore, doctors should be aware that DC could be one of the causes of bone marrow failure in young patients leading to fatal complications like infection and bleeding.

In DC, progressive bone marrow failure can occur; hence we would like to emphasize the importance of proper immunodeficiency management to minimize morbidity and premature mortality in this disease. Our patient has stopped his follow-up in earlier years and has presented with worsening of bone marrow failure complicated by neutropenic fever. Patients also have the responsibility to be aware of their illness and its potential complications, so that they would understand the importance of follow-up and the inter-disciplinary approach to their disease.

### Conclusion

DC is a severe multisystem disorder associated with premature mortality usually due to bone marrow failure. We experienced a patient who was diagnosed as DC with moderate aplastic anemia complicated by neutropenic fever. We believe that by reporting this presentation of the patient would add more information to the existing fund of knowledge. DC is being now studied ever more intensively and this case report can be beneficial not only to the health care professional but also to those suffering from this rare disease.
